# Physiological Oxygen Levels Differentially Regulate Adipokine Production in Abdominal and Femoral Adipocytes from Individuals with Obesity Versus Normal Weight

**DOI:** 10.3390/cells11223532

**Published:** 2022-11-08

**Authors:** Ioannis G. Lempesis, Nicole Hoebers, Yvonne Essers, Johan W. E. Jocken, Kasper M. A. Rouschop, Ellen E. Blaak, Konstantinos N. Manolopoulos, Gijs H. Goossens

**Affiliations:** 1 Institute of Metabolism and Systems Research (IMSR), College of Medical and Dental Sciences, University of Birmingham, Birmingham B15 2TT, UK; 2 Department of Human Biology, NUTRIM School of Nutrition and Translational Research in Metabolism, Maastricht University Medical Centre+, 6229 ER Maastricht, The Netherlands; 3 Centre for Endocrinology, Diabetes and Metabolism, Birmingham Health Partners, Birmingham B15 2TT, UK; 4 Radiotherapy, GROW School for Oncology & Reproduction, Maastricht University Medical Centre+, 6229 ER Maastricht, The Netherlands

**Keywords:** adipose tissue, adipokines, inflammation, body fat distribution, obesity pathophysiology, hypoxia

## Abstract

Adipose tissue (AT) inflammation may increase obesity-related cardiometabolic complications. Altered AT oxygen partial pressure (pO_2_) may impact the adipocyte inflammatory phenotype. Here, we investigated the effects of *physiological* pO_2_ levels on the inflammatory phenotype of abdominal (ABD) and femoral (FEM) adipocytes derived from postmenopausal women with normal weight (NW) or obesity (OB). Biopsies were collected from ABD and FEM subcutaneous AT in eighteen postmenopausal women (aged 50–65 years) with NW (BMI 18–25 kg/m^2^, *n* = 9) or OB (BMI 30–40 kg/m^2^, *n* = 9). We compared the effects of prolonged exposure to different *physiological* pO_2_ levels on adipokine expression and secretion in differentiated human multipotent adipose-derived stem cells. Low *physiological* pO_2_ (5% O_2_) significantly increased leptin gene expression/secretion in ABD and FEM adipocytes derived from individuals with NW and OB compared with high *physiological* pO_2_ (10% O_2_) and standard laboratory conditions (21% O_2_). Gene expression/secretion of IL-6, DPP-4, and MCP-1 was reduced in differentiated ABD and FEM adipocytes from individuals with OB but not NW following exposure to low compared with high *physiological* pO_2_ levels. Low *physiological* pO_2_ decreases gene expression and secretion of several proinflammatory factors in ABD and FEM adipocytes derived from individuals with OB but not NW.

## 1. Introduction

Excess fat mass in obesity poses a major health risk [1]. The research in the past decades has clearly demonstrated that body fat distribution is a better predictor of cardiometabolic complications than total fat mass, with abdominal obesity increasing and lower-body (gluteofemoral) fat accumulation conferring relative protection against chronic cardiometabolic diseases [2,3,4,5,6]. This seems related to the distinct functional properties of these different AT depots. Many studies in rodents and humans have shown that AT dysfunction in obesity is characterised by adipocyte hypertrophy, mitochondrial dysfunction, reactive oxygen species (ROS) production, impaired lipid metabolism, reduced blood flow, and inflammation, together contributing to an increased risk of developing cardiometabolic diseases and cancer [6,7,8,9,10,11].

The AT microenvironment impacts metabolic and inflammatory processes [8,9]. We, and others, have previously demonstrated that AT oxygen partial pressure (pO_2_), which is determined by the balance between local oxygen supply (determined by adipose tissue blood flow) and consumption (primarily mitochondrial oxygen consumption), may be an important determinant of the AT phenotype and whole-body insulin sensitivity [9,12,13,14]. Interestingly, differences in adipose tissue blood flow and/or adipose tissue oxygen consumption between individuals with normal weight and obesity, and between upper and lower AT depots, have previously been demonstrated [9,10,12,15,16]. Although AT pO_2_ is reduced in rodent models of obesity [17,18,19], conflicting findings on AT pO_2_ have been reported in humans [9,20,21,22,23,24]. We have previously shown that AT pO_2_ was higher in individuals with obesity and was positively associated with AT gene expression of proinflammatory markers and whole-body insulin resistance [22,25]. Moreover, we found that AT pO_2_ was lower in femoral than in abdominal subcutaneous AT in women with obesity [16].

The normal *physiological* range of AT pO_2_ in human AT is ~3–11% O_2_ (~23–84 mmHg) [9,21,22,23,25]. Therefore, the outcomes of experiments comparing the effects of pO_2_ below and well above these *physiological* levels should be interpreted with caution, because the results may not directly translate to the human in vivo situation [9]. Several in vitro studies have demonstrated that the expression and secretion of many adipokines are sensitive to changes in pO_2_ levels, as extensively reviewed [9,26]. Most of these studies have shown that acute exposure to severe, non-physiological hypoxia (1% O_2_ for 1–24 h) induces a proinflammatory expression and secretion profile in (pre)adipocytes, while prolonged exposure to mild *physiological* hypoxia (5% O_2_ for 14 days) seems to elicit a different adipokine expression/secretion profile [9,16,27]. Recently, we found that prolonged exposure to low *physiological* hypoxia decreased proinflammatory gene expression in abdominal and femoral adipocytes derived from women with obesity [16]. The metabolic and inflammatory responses to changes in the AT microenvironment may differ between individuals and AT depots. Thus, oxygen levels might exert distinct effects on AT function in people with different adiposity and in different AT depots. Importantly, however, studies investigating the impact of altered pO_2_ levels on the inflammatory phenotype of adipocytes derived from people with normal weight and obesity are lacking.

Therefore, the aim of the present study was to investigate the impact of prolonged exposure to various *physiological* oxygen levels on gene expression and secretion of inflammatory factors within upper and lower body differentiated human multipotent adipose-derived stem (hMADS) cells derived from women with normal weight or obesity.

## 2. Materials and Methods

### 2.1. Upper and Lower Body Adipose Tissue Biopsies

Paired abdominal (ABD) and femoral (FEM) subcutaneous AT needle biopsies were obtained from eighteen postmenopausal women (aged 50–65 years) with normal weight (NW: BMI 18–25 kg/m^2^, *n* = 9) or obesity (OB: BMI 30–40 kg/m^2^, *n* = 9) (Table 1). The U.K. Health Research Authority National Health System Research Ethics Committee approved the present study (approval no. 18/NW/0392). Briefly, the biopsy specimens (up to ∼1 g) were collected 6 to 8 cm lateral from the umbilicus (ABD AT) and from the anterior aspect of the upper leg (FEM AT) under local anaesthesia (1% lidocaine) after an overnight fast. Samples were immediately rinsed with sterile saline, and visible blood vessels were removed with sterile tweezers. Isolation of hMADS cells followed, as described before [16].

### 2.2. Human Primary Adipocyte Experiments

Human multipotent abdominal (ABD) and femoral (FEM) adipose-derived stem cells, an established human white adipocyte model [28], were seeded at a density of 2000 cells/cm^2^ and kept in proliferation medium for seven days. Thereafter, these cells were differentiated under different *physiological* O_2_ levels (10% O_2_, high *physiological* pO_2_; 5% O_2_, low *physiological* pO_2_) [9,16,22,29] as well as standard laboratory conditions (room air, 21% O_2_) for 14 days. Gas mixtures were refreshed every 8 h (to maintain variation <0.1% O_2_), whereas the medium was refreshed three times per week. 

### 2.3. Adipocyte Gene Expression 

Total RNA was extracted from hMADS cells using TRIzol reagent (Invitrogen, Breda, The Netherlands), and SYBR-Green-based real-time PCRs were performed to assess the gene expression of leptin, dipeptidyl-peptidase (DPP)-4, interleukin (IL)-6, plasminogen activator inhibitor (PAI)-1, adiponectin, tumour necrosis factor (TNF)α, and monocyte chemoattractant protein (MCP)-1; the adipocyte differentiation markers peroxisome proliferator-activated receptor γ (PPARγ), CCAAT-enhancer binding protein α (C/EBPα), fatty acid synthase (FAS), and perilipin 1 (PLIN1); as well as the hypoxia markers glucose transporter 1 (GLUT1), Bcl-2 interacting protein 3 (BNIP3), and vascular endothelial growth factor A (VEGFA) using an iCycler (Bio-Rad, Veenendaal, The Netherlands). Results were normalised to 18S ribosomal RNA.

### 2.4. Adipokine Secretion 

The medium of the hMADS cells was collected over 24 h, from day 13 (after replacement of medium) to day 14 of differentiation, to determine the secretion of adipokines using high-sensitive ELISAs (leptin and DPP-4 from R&D Systems, Inc., Minneapolis, MN, USA; IL-6 and MCP-1 from Diaclone SAS, Besancon Cedex, France; adiponectin and PAI-1 from BioVendor–Laboratorni medicina a.s., Brno, Czech Republic). If necessary, samples were diluted with the dilution buffer provided by the manufacturer prior to the assay, which was performed in duplicates according to the manufacturer’s instructions.

### 2.5. Statistical Analyses

Data are presented as mean ± SEM. The effects of exposure to different oxygen levels on adipocyte gene expression and adipokine secretion were analysed using one-way ANOVA or the Friedman test when data were not normally distributed, followed by post hoc comparison using Student’s paired t-tests or the Wilcoxon signed-rank test in case of skewed data. GraphPad Prism version 8 for Windows (GraphPad Software, San Diego, CA, USA) was used to perform statistical analyses. *p* < 0.05 was considered statistically significant.

## 3. Results

### 3.1. The Effects of Oxygen Partial Pressure on Adipocyte Gene Expression

**Figure 1:**
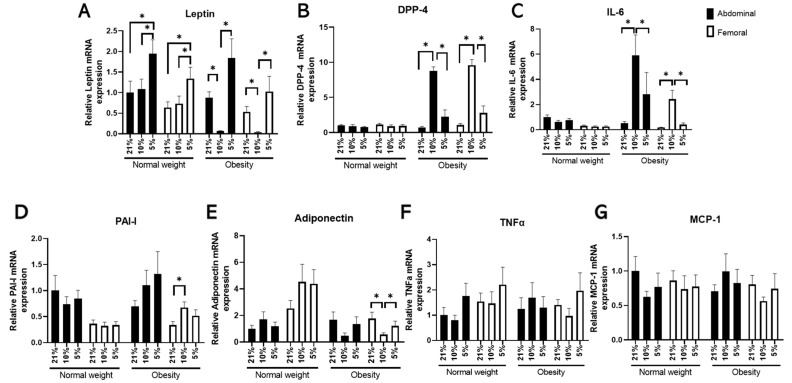
Adipokine and inflammatory markers’ gene expression in hMADS cells following differentiation under different pO_2_s (21% vs. 10% vs. 5% O_2_) (*n* = 9 paired samples): (**A**) *leptin*, (**B**) *dipeptidyl-peptidase (DPP)-4*, (**C**) *interleukin (IL)-6*, (**D**) *plasminogen activator inhibitor (PAI)-1*, (**E**) *adiponectin*, (**F**) *tumour necrosis factor (TNF)α*, and (**G**) *monocyte chemoattractant protein (MCP)-1*. Data are expressed as mean ± SEM. * *p* < 0.05.

The exposure of differentiated hMADS cells derived from ABD and FEM AT to different pO_2_ levels induced distinct gene expression patterns. Specifically, exposure to low *physiological* pO_2_ (5% O_2_) increased *leptin* expression compared with exposure to high *physiological* pO_2_ (10% O_2_) or room air (21% O_2_) in differentiated ABD and FEM hMADS derived from individuals with NW as well as OB (all *p* < 0.01, Figure 1A). Furthermore, low *physiological* pO_2_ markedly reduced the gene expression of the proinflammatory factors *DPP-4* and *IL-6* in both ABD and FEM differentiated hMADS derived from donors with OB (all *p* < 0.01) but not NW compared with high *physiological* pO_2_ (Figure 1B,C). Low *physiological* pO_2_ levels did not significantly alter the gene expression of *PAI-1*, *TNFα*, or *MCP-1* in differentiated ABD and FEM hMADS derived from NW and OB individuals (Figure 1D–G), except for a modest but significant (*p* = 0.041) increase in adiponectin gene expression in FEM differentiated hMADS derived from individuals with obesity (Figure 1E). In addition, high *physiological* AT pO_2_ (10% O_2_) increased the *PAI-1* (*p* = 0.005) and reduced the *adiponectin* expression (*p* = 0.010) in FEM differentiated hMADS derived from individuals with OB compared with those at 21% O_2_ exposure. As expected, exposure to *physiological* oxygen levels, i.e., lower oxygen levels compared with standard laboratory conditions, increased the gene expression of the classical hypoxia markers *GLUT1* and *VEGFA*, and, to a lesser extent, increased that of *BNIP3* (Figure S1A–C). Furthermore, exposure to low *physiological* oxygen levels (5% O_2_) did not alter the gene expression of adipocyte differentiation markers compared with room air (21% O_2_) in differentiated hMADS derived from individuals with NW as well as OB (Supplemental Figure S1D–G). In the differentiated hMADS derived from individuals with OB, the gene expression of *PPARγ*, *C/EBPα*, and FAS was lower, and expression of *PLIN1* higher, following exposure to 5% compared with 10% O_2_.

### 3.2. The Effects of Oxygen Partial Pressure on Adipokine Secretion

**Figure 2:**
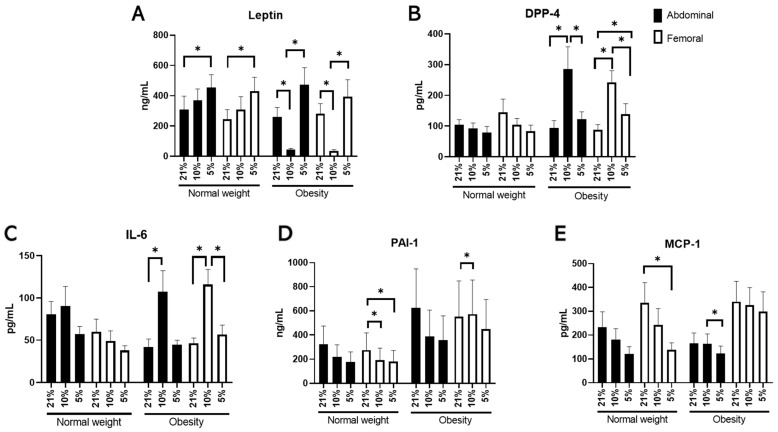
Adipokine and inflammatory markers’ secretion in hMADS cells following differentiation under different pO_2_s (21% vs. 10% vs. 5% O_2_) (*n* = 9 paired samples). (**A**) Leptin, (**B**) dipeptidyl-peptidase (DPP)-4, (**C**) interleukin (IL)-6, (**D**) plasminogen activator inhibitor (PAI)-1, and (**E**) monocyte chemoattractant protein (MCP)-1. Data are expressed as mean ± SEM. * *p* < 0.05.

Next, we investigated whether exposure to different pO_2_ levels elicited functional changes in adipokine secretion from differentiated ABD and FEM hMADS. We found that adipokine secretion from both differentiated ABD and FEM hMADS was significantly affected by changes in oxygen availability (Figure 2). Specifically, low *physiological* pO_2_ (5% O_2_) exposure increased leptin secretion in differentiated ABD and FEM hMADS derived from individuals with OB compared with exposure to high *physiological* pO_2_ (10% O_2_: ABD, *p* = 0.009; FEM, *p* = 0.021), and in differentiated ABD and FEM hMADS derived from individuals with NW compared with exposure to room air (21% O_2_: ABD, *p* = 0.014; FEM, *p* = 0.006) (Figure 2A). Furthermore, DPP-4 secretion was significantly lower following exposure to low (5% O_2_) compared with high (10% O_2_) *physiological* pO_2_ in differentiated ABD (*p* = 0.027) and FEM hMADS (*p* = 0.004) and IL-6 secretion in differentiated FEM hMADS only (*p* = 0.007), derived from donors with OB but not NW (Figure 2B,C). Moreover, low *physiological* pO_2_ (5% O_2_) reduced MCP-1 secretion (*p* = 0.030) but did not alter PAI-1 secretion from differentiated ABD hMADS derived from individuals with OB compared with 10% O_2_ (Figure 2D,E). Finally, low *physiological* pO_2_ (5% O_2_) reduced both MCP-1 (*p* = 0.028) and PAI-1 (*p* = 0.003) secretion from differentiated FEM hMADS derived from donors with NW compared with 21% O_2_ (Figure 2D,E). Adiponectin secretion was not detectable, and these data are therefore not reported.

## 4. Discussion

In the present study, we investigated the impact of oxygen tension on adipokine gene expression and secretion in differentiated human multipotent ABD and FEM adipose-derived stem cells from women with NW or OB. Here, we demonstrate that low *physiological* pO_2_ decreased gene expression and secretion of the proinflammatory factors *DDP-4* and *IL-6* in both differentiated ABD and FEM hMADS derived from individuals with OB, while these responses were not present in differentiated hMADS cells from NW individuals. Our findings highlight that the changes in pO_2_ within the human *physiological* range in the adipocyte microenvironment contribute to alterations in the AT inflammatory phenotype and that these effects may differ between individuals with normal weight and obesity.

To determine whether the amount of oxygen present in the AT microenvironment affects the gene expression of adipokines, we exposed differentiating hMADS cells from ABD and FEM AT to low (5%) and high (10%) *physiological* pO_2_ levels in human AT [9,16,21,22,23,24]. As expected, low *physiological* pO_2_ levels increased the gene expression of several hypoxia markers. Strikingly, we show for the first time that low *physiological* pO_2_ during adipogenesis consistently decreased the expression and secretion of the proinflammatory markers IL-6 and DPP-4 in both differentiated FEM and ABD hMADS derived from individuals with OB, but not NW. Moreover, the present data suggest that these cells maintain a memory of origin (i.e., a normal-weight or obese microenvironment) in vitro, even after 14 days of exposure to the same experimental conditions. In agreement with our findings, we have previously reported that in vivo ABD AT pO_2_ was positively associated with the AT gene expression of several proinflammatory markers [22] and that low *physiological* pO_2_ exposure reduced the gene expression of IL-6 and DPP-4 in adipocytes derived from women with obesity [16]. In addition, the present results show that low *physiological* pO_2_ levels consistently increased leptin gene expression and secretion in differentiated ABD and FEM hMADS derived from donors with NW or OB. Leptin is an important regulator of appetite and energy expenditure, providing important feedback in relation to energy storage in the body through the hypothalamus, and is involved in multiple *physiological* processes such as the regulation of immunity [9,30,31,32]. Changes in leptin secretion due to altered oxygen tension in the AT microenvironment may thus affect these processes. Notably, pO_2_-induced alterations in adipokine gene expression were paralleled by comparable changes in adipokine secretion. Importantly, the modest effects of pO_2_ levels on adipocyte differentiation, if present at all, do not seem to explain the observed changes in adipokine expression and secretion, exemplified by the opposing effects of low pO_2_ on the expression and secretion of leptin and the proinflammatory markers Il-6 and DPP-4. Famulla et al. [27] previously showed increased DPP-4, adiponectin, and IL-6 secretion following prolonged exposure to high *physiological* pO_2_ (10% O_2_), while low *physiological* pO_2_ (5% O_2_) tended to reduce the secretion of adiponectin. These differences between studies may at least partly be explained by differences in donor characteristics.

A strength of the present study is the paired comparisons between differentiated adipose-derived multipotent stem cells derived from ABD and FEM AT of individuals with NW and OB. Previous studies examining the effects of pO_2_ levels on adipocyte inflammation have either used cell lines, adipose-derived multipotent stem cells from a single donor, or a pool of stem cells obtained from different donors. Because our findings demonstrate that the impact of changes in the AT microenvironment (i.e., different *physiological* pO_2_ levels) on adipokine expression and secretion depends on the characteristics of the donors, future studies in the field of AT biology should take this ‘*memory-of-origin effect*’ into account. Secondly, in contrast to many studies showing that acute exposure to severe (non-physiological) hypoxia evokes a proinflammatory response in murine and human (pre)adipocytes [13,14], we aimed to mimic *physiological* in vivo conditions in terms of pO_2_ levels as well as the prolonged exposure duration in the present study. This study also has some limitations. We examined the effects of various oxygen levels in cells derived from postmenopausal women. Therefore, our findings cannot be translated to other subgroups of the population such as men or individuals of different age. Furthermore, we used a targeted approach to examine the gene expression and secretion of several adipokines. Future studies using an untargeted approach (e.g., microarray analysis, RNA sequencing, or proteomics) are warranted.

## 5. Conclusions

In conclusion, the present findings demonstrate that *physiological* oxygen levels regulate adipokine expression and secretion in differentiated ABD and FEM hMADS. Differentiated hMADS cells derived from women with OB display lower expression and secretion of several (proinflammatory) adipokines at low (5% O_2_) compared with high (10% O_2_) *physiological* oxygen tension. Except for the effects on leptin expression, no significant effects of low compared with high *physiological* oxygen levels were observed in differentiated hMADS cells derived from individuals with NW. Our findings thus indicate that pO_2_ levels alter the expression and secretion of several adipokines in differentiated human ABD and FEM hMADS, and that donor characteristics determine experimental outcomes. This has important implications for future mechanistic in vitro studies in the field of AT biology. For example, the outcomes of studies in which the effects of certain interventions on adipocyte inflammation and related biological mechanisms are investigated may depend on the microenvironmental oxygen tension. Furthermore, our findings highlight that it is important to report detailed the characteristics of the cell donor(s) in studies examining human adipocyte biology.

## Figures and Tables

**Table 1:** Subjects’ characteristics.

Parameter	Normal Weight (*n* = 9)	Obesity (*n* = 9)	*p* Value
Age (years)	56.7 ± 1.8	56 ± 1.3	0.566
BMI (kg/m^2^)	22.8 ± 0.4	34.8 ± 1.3	<0.001
Waist circumference (cm)	79.4 ± 3.1	105.2 ± 3.8	<0.001
Hip circumference (cm)	94.4 ± 2.8	119.9 ± 4.8	<0.001
Waist-to-hip ratio	0.84 ± 0.02	0.88 ± 0.04	0.127
Visceral fat mass (g)	402.5 ± 118	1,325 ± 153.3	0.003
Abdominal fat mass (kg)	10.01 ± 1.48	24.4 ± 2.37	<0.001
Leg fat mass (kg)	7.67 ± 0.86	16.03 ± 1.43	0.001
Fasting glucose (mmol/L)	4.91 ± 0.10	5.10 ± 0.23	0.416
2-hour glucose (mmol/L)	4.90 ± 0.34	4.70 ± 0.33	0.684
Fasting insulin (pmol/L)	28.40 ± 5.80	43.30 ± 10.20	0.202
HOMA2 IR	0.46 ± 0.10	0.72 ± 0.20	0.187
SBP (mmHg)	119.6 ± 4.4	133.0 ± 3.1	0.039
DBP (mmHg)	73.6 ± 4.3	81.7 ± 1.9	0.153

BMI, body mass index; DBP, diastolic blood pressure; HOMA2 IR, Homeostasis Model Assessment 2 Insulin Resistance; SBP, systolic blood pressure. Data are mean ± SEM.

## Data Availability

Data presented in this manuscript are available from the corresponding author on reasonable request.

## Supplementary Materials

The following supporting information can be downloaded at: https://www.mdpi.com/article/10.3390/cells11223532/s1, Figure S1: Adipocyte differentiation and hypoxia markers’ gene expression in adipose tissue-derived mesenchymal stem cells following differentiation under different pO_2_s (21% vs. 10% vs. 5% O_2_).
