# Supplementary material for: Physiological Oxygen Levels Differentially Regulate Adipokine Production in Abdominal and Femoral Adipocytes from Individuals with Obesity Versus Normal Weight
Source: Cells. 2022 Nov 8;11(22):3532. doi: 10.3390/cells11223532 (PMC9688196; doi:10.3390/cells11223532)
Supplement: Supplementary file 1 [file cells-11-03532-s001.zip › cells-1980981-supplementary.pdf]

cells

MDPI

Supplementary Materials

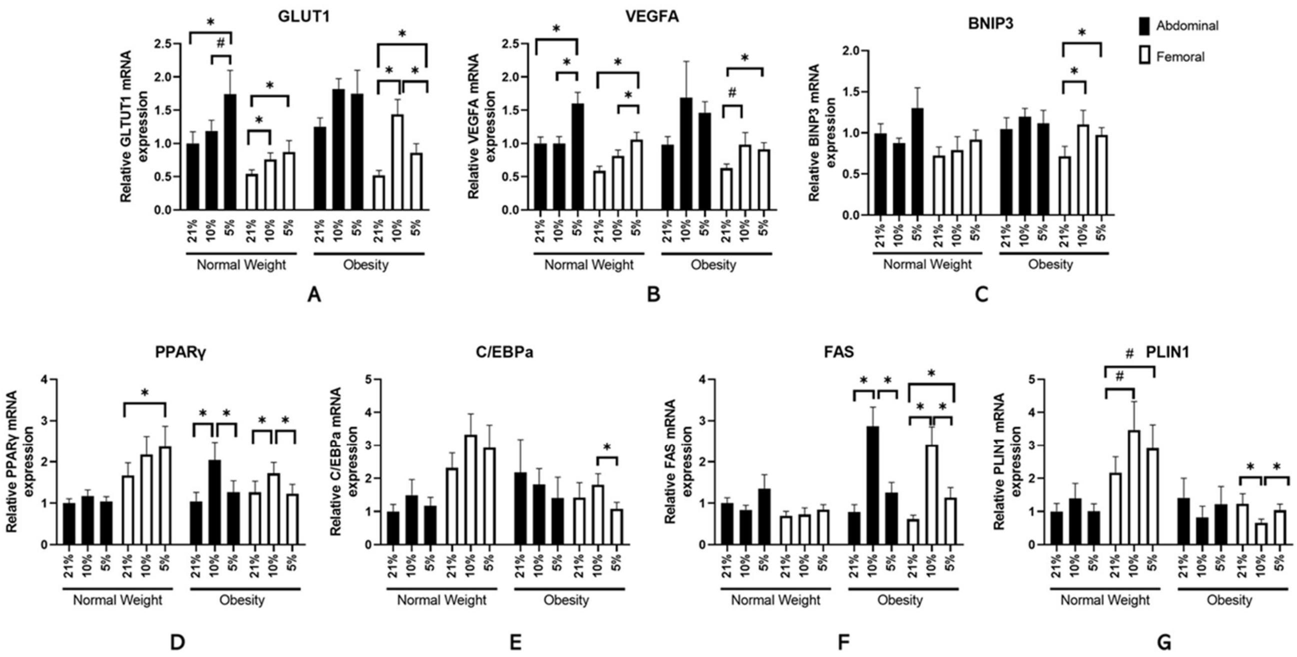

Figure S1: Adipocyte differentiation and hypoxia markers gene expression in adipose tissue-derived mesenchymal stem cells following differentiation under different pO2s (21% vs. 10% vs. 5% O_2). Panel A: GLUT1, glucose transporter 1; B: VEGFA, vascular endothelial growth factor A; C: BNIP3, Bcl-2 interacting protein 3; D: PPARγ, peroxisome proliferator-activated receptor γ; E: C/EBPα, CCAAT-enhancer binding protein α; F: FAS, fatty acid synthase; G: PLIN1, Perilipin 1. Data are expressed as mean ± SEM. * $p < 0.05$, # $p < 0.01$
